# A mixed-methods study to evaluate the effectiveness and cost-effectiveness of aerobic exercise for primary dysmenorrhea: A study protocol

**DOI:** 10.1371/journal.pone.0256263

**Published:** 2021-08-16

**Authors:** Priya Kannan, Kwok-Kuen Cheung, Benson Wui-Man Lau, Lin Li, Huijun Chen, Fenghua Sun

**Affiliations:** 1 Department of Rehabilitation Sciences, The Hong Kong Polytechnic University, Hung Hom, Hong Kong; 2 Department of Applied Social Sciences, The Hong Kong Polytechnic University, Hung Hom, Hong Kong; 3 Saw Swee Hock School of Public Health, National University of Singapore, Singapore, Singapore; 4 Department of Health and Physical Education, Education University of Hong Kong, Hung Hom, Hong Kong; Prince Sattam Bin Abdulaziz University, College of Applied Medical Sciences, SAUDI ARABIA

## Abstract

**Background and purpose:**

Several studies have evaluated the effects of high-intensity aerobic training (HIAT) on pain severity and quality of life (QoL) among women with primary dysmenorrhea. However, to date, no studies have evaluated the effectiveness of HIAT on academic performance or absenteeism or examined the cost-effectiveness of HIAT relative to other treatments in women with primary dysmenorrhea. Furthermore, the mechanisms underlying aerobic exercise-induced analgesia in primary dysmenorrhea remain unclear. The objectives of this study are to: (1) evaluate the effects of HIAT on absenteeism and academic performance among university students, (2) identify the underlying mechanisms associated with aerobic exercise-induced analgesia in primary dysmenorrhea, and (3) determine the cost-effectiveness of HIAT compared with a wait-list control (WLC) group receiving usual care.

**Methods:**

A sequential, embedded, mixed-methods study design, including a crossover, randomised controlled trial (RCT) and semi-structured focus groups, will be conducted alongside an economic evaluation. A total of 130 women aged 18–24 years will be randomised into either HIAT (n = 65) or wait-list control (n = 65) groups. Primary outcomes will include average pain intensity, absenteeism from university, and academic performance. Primary mediators will include salivary progesterone and prostaglandin F2α levels. Outcome and meditator variables will be assessed at baseline and post-treatment, at 12 and 28 weeks. An economic analysis will be conducted from the societal and healthcare perspective of Hong Kong. Semi-structured focus groups will be conducted at 32 weeks. Of the 130 participants included in the RCT, 70 will be included in the focus groups.

**Statistical analysis:**

All statistical analyses will be performed on an intention-to-treat basis, using SPSS (version 24). Preliminary analysis using an independent samples *t*-test and a two-sided, unpaired Student’s *t*-test will be performed to exclude carryover effects and identify within-participant differences in outcome variables between the study periods, respectively. Treatment effects will be evaluated using analysis of variance via a mixed-effects model with fixed effects for intervention, period, and sequence. In all models, random effects will include the participants nested within the sequence as a sampling cluster. The mediation effects will be assessed using the Sobel test. The EQ-5D responses will be converted into utility scores to estimate the gain or loss of quality-adjusted life-years. Seemingly unrelated regression analyses will be used to estimate the total cost differences and effect differences. Qualitative data will be analysed using the process of thematic analysis.

## Introduction

Primary dysmenorrhea is a debilitating condition that affects nearly half of all menstruating women worldwide [1]. A cross-sectional survey performed in 2013 in Hong Kong (HK) found that among 240 18–25-year-old female university students, the prevalence of dysmenorrhea was 80% [2]. Primary dysmenorrhea has been linked to regular educational absenteeism [3], and the class attendance rate among women has been reported to decrease by 29%–50% during menstruation [4]. Recurrent absenteeism has negative impacts on young women, decreasing the total contact time for learning, which may affect the quality of the educations they receive [3]. Studies that have examined the impacts of primary dysmenorrhea on daily life have reported that adolescent women with primary dysmenorrhea experience a reduced ability to concentrate, educational disruptions [2], and lower academic performance [5]. The effects of primary dysmenorrhea-associated pain extend beyond individual women to society, resulting in regular work absenteeism [5].

The pathogenesis of primary dysmenorrhea has been linked to abnormally elevated levels of prostaglandin secretion [1,6]. Abnormally high prostaglandin levels cause frequent and dysrhythmic uterine contractions, resulting in ischaemia and hypoxia of the uterus, which are considered to be major contributors to primary dysmenorrhea-associated pain [1,7]. Aetiological studies have reported that prostaglandin production is regulated by progesterone, with prostaglandin and progesterone displaying an inverse relationship [1,6,8].

Aerobic exercise has been associated with a reduced prevalence of primary dysmenorrhea and a decrease in related symptoms in several studies [9–12]. Our preliminary studies [13,14] showed that primary dysmenorrhea-associated pain intensity was reduced by engaging in 30 minutes of treadmill-based high-intensity aerobic training (HIAT), performed three days a week at 70%–85% of the age-adjusted maximum heart rate (MHR). We found that one month of supervised, treadmill-based HIAT, supplemented with six months of unsupervised HIAT, was effective for decreasing pain intensity and improving QoL and physical function relative to usual care [13]. However, no study has yet evaluated the effects of exercise, specifically HIAT, on absenteeism and academic performance among university students or the cost-effectiveness of HIAT for the management of primary dysmenorrhea. Another knowledge gap in this area concerns questions regarding the physiological mechanisms that underlie the beneficial effects of aerobic exercise-induced pain relief in primary dysmenorrhea. No study has yet identified the physiological mechanisms that mediate the beneficial effects of aerobic exercise-induced pain relief in primary dysmenorrhea. Therefore, we conducted a pilot study [15] to explore the mechanisms through which HIAT exerts beneficial effects on pain associated with primary dysmenorrhea. Our pilot study findings indicated a trend towards increased progesterone levels and decreased prostaglandin levels in the HIAT group compared with the control group, suggesting that aerobic exercise may act to reduce primary dysmenorrhea-associated pain through effects on these mediators [15]. Here, we propose an adequately powered, full-scale study to (1) evaluate the effects of HIAT on absenteeism and academic performance among university students, (2) identify the underlying mechanisms associated with aerobic exercise-induced analgesia in primary dysmenorrhea, and (3) determine the cost-effectiveness of HIAT compared with a wait-list control (WLC) group receiving usual care.

### Hypotheses

The proposed crossover randomised controlled trial (RCT) will test the following three hypotheses. (1) Engaging in HIAT for 12 weeks will lead to significant reductions in pain intensity and absenteeism and improve academic performance compared with baseline levels. (2) Pain improvements will be mediated by increased progesterone levels, resulting in decreased prostaglandin F2α (PGF2α) levels. (3) HIAT will display superior cost-effectiveness compared with usual care (WLC) for primary dysmenorrhea treatment.

## Materials and methods

### Research design, recruitment, and study setting

A sequential, embedded, experimental mixed-methods design, including a crossover RCT and semi-structured focus groups, will be conducted alongside an economic evaluation. In this experimental model, qualitative data will be embedded within the quantitative methodology [16]. Qualitative data will be collected after the intervention to follow up on the experiences of the participants during the intervention. A mixed-methods approach will be considered because the combination of qualitative and quantitative methods can contribute to interdisciplinary and comprehensive research evidence [17]. A crossover design will be adopted to minimise the effects of confounders and inter-individual variability (each participant serves as their own control) [18]. Ethics approval for the trial was obtained from the Human Subjects Ethics Application Review System of the HK Polytechnic University (PolyU; Ref. No.: HSEARS20201109002). Any changes to the trial protocol, including any modifications of the study objectives, design, population, sample sizes, or procedures, will be approved by the institutional review board prior to implementation. Research personnel will explain all study procedures to all participants, and written informed consent will be obtained prior to their enrolment in the study. This study protocol is registered with ClinicalTrial.gov (Ref. No. NCT04665661; date of registration: 7 December 2020). All items from the World Health Organization Trial Registration Data Set can be found at https://clinicaltrials.gov/ct2/show/record/NCT04665661. This trial will include: (1) a two-step screening phase for eligibility; (2) a baseline assessment (time-point [T1]); (3) 12 weeks of supervised treadmill training for the HIAT group; (4) post-treatment outcome assessments and economic evaluations at 12 weeks (T2); (5) a four-week washout period, followed by the crossover of the HIAT and WLC groups; (6) 12 weeks of supervised treadmill training for the WLC group; (7) post-treatment outcome assessments at 28 weeks (T3); and (8) semi-structured focus groups at 32 weeks. Potential participants will be recruited via advertisements (study flyer) distributed at the HK PolyU and the Education University of HK campuses and throughout the local community and by word-of-mouth. This study will be conducted at the Department of Rehabilitation Sciences, HK PolyU.

### Sample size

G-Power analysis [19] (3.1.9.2), set for an F-test (analysis of variance [ANOVA]), was used to estimate the sample size required to reliably test the study hypotheses. Based on estimated changes in progesterone levels from our pilot trial (***d*** = 0.36) [15], the sample size required to detect differences in progesterone levels with 90% power at the 5% significance level was calculated to be 100 (50 per group). Assuming a 30% drop-out rate, 130 participants (65 per group) will be recruited for the crossover RCT. Using estimates of change in pain levels from our previous study [15] (*d* = 0.40), the required sample size is 54 per group (power = 90%, alpha = 0.05 and 30% attrition rate). However, we plan to enrol many more than 54 participants per group (i.e., 65 per group) in order to ensure that we have adequate power to test all three of the study hypotheses.

### Participants

Eligibility criteria are consistent with the guidelines for the diagnosis of primary dysmenorrhea as established by the Society of Obstetrics and Gynecologists of Canada [20]. Inclusion criteria include (1) 18–24 years of age; (2) non-pregnant; (3) experiencing regular menstrual cycles, with cycle lengths between 24 and 30 days; (4) experiencing an average menstrual pain intensity equal to or greater than 5 on a 0–10 Numerical Rating Scale (NRS); and (5) scoring low (<600 metabolic equivalent tasks [MET]/week) on the short-form of the International Physical Activity Questionnaire (sf-IPAQ). The sf-IPAQ is reported to be a valid [21] and reliable [22] tool for assessing and monitoring physical activity. Exclusion criteria include (1) the use of oral contraceptive pills, hormonal therapy, or intrauterine devices; (2) the use of over-the-counter analgesics during menstruation to treat dysmenorrhea-associated pain but experiencing no pain relief in response to those analgesics; and (3) participation in any formal exercise programme.

### Randomisation and blinding

An individual who is not involved in the study will develop a randomisation schedule (computer-generated) and prepare 130 sealed, opaque envelopes for allocation concealment. The sealed opaque envelopes will contain the group name (HIAT or WLC) and a personal identification number (PIN). Following the baseline assessment, participants will select an envelope containing the details of their treatment allocation. The nature of the intervention is such that neither the participants nor the therapist can be blinded to the treatment allocation. A blinded examiner will assess all study outcomes and collect saliva samples for the estimation of primary and secondary mediators. Enzyme-linked immunosorbent assays (ELISAs) will be performed in a blinded manner by research personnel not involved in the assessment of study outcomes. Data analysis will be conducted in a blinded manner, using coded groups (Group A and Group B). The group code will only be revealed after all analyses have been completed.

### Procedure

Potential participants will undergo a two-step screening process. The first step will be conducted via telephone interview, using an eligibility checklist. Potentially eligible women will then be invited to an in-person screening session on the first day of their next menstrual period. During this session, women will complete the screening questionnaire and sf-IPAQ to determine eligibility and assess baseline physical activity levels. Written informed consent and anthropometric measurements will be acquired. Baseline assessments of the primary and secondary outcomes, information regarding the costs and absenteeism associated with each individual’s previous three menstrual cycles, and saliva samples will be collected. Participants will then be randomised into HIAT or WLC groups. Participants in the HIAT group will receive supervised treadmill training for the first 12 weeks of the trial. To minimise carryover effects, a four-week washout period will be observed for all study participants before cross-over. During the washout period, the HIAT group will be asked to discontinue exercise, and both groups will continue to manage pain as usual (i.e. with analgesics). After the four-week washout period, the cross-over period will begin during Week 16, and the participants who were initially randomised to the WLC group will receive the HIAT regimen from Weeks 16 to 28. Participants who were initially randomised to the HIAT group will be instructed not to engage in exercise during the remainder of the study period (i.e. Weeks 16–28). Participants will be permitted to use analgesics for menstrual pain, as needed, throughout the study and will be instructed to record the type and dose (in milligrams) of analgesics consumed in an electronic diary (e-diary). A diagnosis of primary dysmenorrhea can be confirmed by collecting detailed history [23]; therefore, a pelvic examination/ultrasound will not be conducted.

### Saliva collection

Saliva will be collected as described in a previous study [24]. All participants will receive salivary kits and specimen bags coded with participant details (name, PIN, and group allocation) and written instructions for saliva collection procedures. Training will be provided to all participants for the collection of citric acid-stimulated saliva (approximately 5 mL). The home collection of saliva is convenient, cost-effective, and can be performed easily and safely without assistance [25]. Participants will be instructed to collect saliva immediately after the start of menstruation and two hours following the start of menstruation, to store samples in a freezer [24]. Research personnel will transport the samples to the lab for further processing.

### Intervention

The exercise intervention will last for 24 weeks (12 weeks for the HIAT group and 12 weeks for the WLC group) at PolyU. Supervised treadmill (Zebris FDM-T) training at PolyU will begin on the first day of the second menstrual period (baseline assessments will be completed during the first menstrual period). Exercise sessions at PolyU will be instructed and supervised by a registered physiotherapist. Using this supervisory method, the participant-to-research personnel ratio will be 1:1. The rationale for providing supervised sessions is to increase adherence to study interventions because, in our previous RCT^9^, adherence was higher for supervised sessions than for unsupervised home exercise sessions. MHR will be calculated using the conventional age-predicted formula (220 − age). To achieve the target HR, the speed of the treadmill will be gradually increased, based on the participants’ HR, which will be displayed on the treadmill, and the rating of perceived exertion (RPE). Because the current proposed study does not involve graded, incremental exercise, graded exercise testing (such as VO_2max_ evaluation) will not be performed.

#### HIAT

Women will perform treadmill-based aerobic exercise for three days a week, at 70%–85% of MHR for 30 minutes and at a perceived exertion level of 14–16, based on the Borg RPE scale. This range [26] is considered to represent HIAT. Aerobic training will be preceded by warm-up exercises for 10 minutes and followed by cool-down exercises for 10 minutes, at a perceived exertion of 11.0 (according to the Borg RPE). These exercise parameters are based on our previous studies [13,14] and the American College of Sports Medicine guidelines [27].

#### WLC

Women in the WLC group will be instructed to continue with their usual activities and manage their pain as normal (i.e. with analgesics).

### Safety and adverse events

The anticipated adverse events include falls from the treadmill, sprained joints resulting from falls, or muscle strains. To minimise these risks, a registered physiotherapist will continuously monitor the participants while they use the treadmill. Serious adverse events are highly unlikely to occur as the intervention being studied is a low-risk intervention. Additionally, in our previous studies [13,14] examining the effects of aerobic exercise for women with primary dysmenorrhea no adverse events were identified or reported.

### Outcome measures

#### Primary mediators

Primary mediators will include progesterone and PGF2α levels in saliva. Salivary measures provide a reliable and non-invasive method for assessing unbound steroid hormones in humans [28]. Salivary progesterone levels have been significantly correlated with plasma levels during the menstrual cycle and reflect free serum progesterone levels [28]. Previous studies have identified elevated levels of PGF2α in saliva during menstruation [24] and have reported that saliva could be used as an important source of biomarkers, such as PGF2α [29].

#### Primary outcomes

Pain intensity during the last 24 hours of menstruation will be assessed using the 0–10 NRS [30], with 0 representing ‘no pain’ and 10 representing the ‘worst imaginable pain’.

Absenteeism from the university will be recorded using an e-diary. Access to a prospective e-diary will be provided to each participant for recording university absenteeism. E-diaries have been are reported to be highly acceptable and feasible for use in research [31]. Significantly increased compliance and accuracy have been reported for the use of e-diary recording compared with traditional paper diaries [31].

Academic performance will be measured using the self-reported 20-item academic performance questionnaire, which was developed to measure the impacts of menstrual symptoms on academic performance [32].

#### Secondary outcomes

The 0–10 visual analogue scale will be used to measure the impact of dysmenorrhea on concentration [33], with 0 representing ‘no difficulty concentrating’ and 10 representing ‘maximum difficulty concentrating’.

The 10-item, disease-specific dysmenorrhea daily diary (DysDD; electronic version) will be used to measure the severity of menstrual bleeding, the use of analgesic (number of tablets/pills) to treat pelvic pain, and the impacts of menstrual pain on work/education, physical and social activities, and sleep [34]. The electronic version of DysDD has been reported to be an adequately reliable and valid tool for assessing dysmenorrhea [35]. To minimise burden, participants will be asked to complete the DysDD only on days when their pain rating is non-zero or when rescue analgesics are taken [34,35].

If evidence of extreme ratings is identified in the self-reported outcome measures, research personnel will contact the participant over the phone to obtain more information regarding any major changes in symptoms.

#### Economic evaluation

We will analyse the results of HIAT versus WLC over a 12-week, within-trial period (i.e., at T2) [36]. The cost-effectiveness for the entire 28-week time horizon will also be explored through T3 data observed in the HIAT arm and extrapolated from WLC data [36]. The primary analysis will be conducted from a societal perspective (short-term direct costs; intervention costs; costs unrelated to the intervention [healthcare services and medication costs]; and indirect costs, such as absence from work and impact on productivity due to primary dysmenorrhea) [37]. The secondary analysis will be conducted from a healthcare perspective (direct costs, including intervention costs; and costs of other care, *excluding* absenteeism costs) [37]. Cost and absenteeism data collected at baseline (i.e. for the previous three menstrual cycles) will be used as predictors of future costs in the analysis. The EQ-5D-3L will be used to determine the QoL for the HIAT and WLC groups. The cost per participant will be calculated by multiplying unit costs identified from the HK government standardised national price list and resource quantities [36].

An electronic diary (https://forms.gle/gvoLHAoDqySQFr4GA) will be used to record cost expenditures and work absenteeism. All participants will be provided with access to the cost diary post-randomisation. Participants with limited access to the internet will be provided with printed hard copies of the cost and absenteeism diary, along with stamped and addressed return envelopes. Participants will be instructed to record any forms of medical intervention undertaken during the study period and will be encouraged to record the intervention in the relevant column of the cost diary. In addition, participants will be asked to retain treatment receipts. The treatment receipts will be verified against the relevant entry, and the original receipt will be returned to the participant. Participants will be instructed to complete the diary during the week of menstruation each month. The research team members will be able to access the electronic diary to obtain a summary of completed items for all study participants. A student assistant will contact participants by telephone to acquire additional details for incomplete entries or when participants fail to complete the diary.

#### Treatment fidelity and missed sessions

To optimise adherence to exercise sessions at PolyU, research personnel (student assistant) will contact any participant who has missed an exercise session and attempt to re-schedule that session. Following the baseline assessment, all participants will be provided with information regarding how to access and complete the electronic diaries.

#### Retention plan

To maximise retention, we (1) will obtain the contact information of participants and any friends and relatives who may know their whereabouts and obtain permission to follow up with any listed contact persons for study purposes; (2) will include appointments for data collection in the study schedule; and (3) will schedule supervised exercise at convenient times for participants.

### Qualitative study (semi-structured focus groups)

The qualitative study will provide insight into the participants’ experiences during and attitudes towards the study intervention. During the focus group discussions, attempts will be made to identify the subjective experiences of participation when engaging in HIAT, in addition to participants’ perceptions regarding any changes in menstrual pain, university attendance, or academic performance. Ethical approval for the focus groups will be obtained from the Human Subjects Ethics Committee of PolyU before participant enrolment. Written informed consent to participate in the focus groups will be obtained. A sample size of 50–60 is recommended for qualitative research [38]; therefore, a sample size of 70 participants is planned.

For this component of the research, a computer-generated randomisation schedule will be generated and utilised to identify 70 participants (from the 130 participants included in the RCT), to participate in the focus groups. Invitations to participate in the focus groups will be sent to potential participants at the end of 28 weeks. Invitations will contain contact information, including a phone number, email address, and mailing address, for research personnel. Interested participants will be asked to express interest in participation by phone, email, or post. The recruitment period for the focus group will last for three weeks. A total of seven focus groups will be conducted at 32 weeks post-randomisation. Each group will consist of a random sample of 8–10 participants and will last between 30 and 45 minutes. Focus groups will be conducted in private rooms at PolyU. If the coronavirus disease 2019 pandemic continues, face-to-face seminars will be replaced with virtual meetings (via Zoom) to allow for appropriate social distancing measures. A semi-structured interview technique will be used, in which the discussion will be guided by but not limited to pre-determined (open-ended and probing) questions. At the start of the interview, participants will be assured that their anonymity will be protected through the use of participant code numbers. Two researchers, a moderator and a co-moderator, will conduct the focus groups, and a third research team member will take notes. At the conclusion of each focus group, the co-moderator will present a summary of the discussion to the group, and the participants will be provided with an opportunity to add or modify any comments. All focus groups will be audio recorded (using a digital voice recorder, in addition to taking notes), transcribed, and anonymised.

### Statistical tests and anticipated results

#### Missing data, data preparation, and monitoring

We will implement strategies, such as the digital diary, follow-up phone-call reminders, and providing routine encouragement to continue exercise, to minimise missing data. All statistical analyses will be performed on an intention-to-treat basis using SPSS (version 24). A data monitoring committee will not be required for the currently proposed trial because it does not have any of the following features: major safety concerns, unknown risks, long-term follow-up, or double-blind treatment assignment [39].

#### Hypothesis 1

The two phases that each participant completes during the course of a crossover trial are typically referred to as the two study periods [40]. First, a preliminary test will be performed to exclude carryover effects and evaluate our study for validity [41]. This will be performed by estimating the sum of the values measured during the two treatment periods for each participant and compared across the groups by using an independent samples *t*-test [40]. Second, the within-participant differences in outcome variables between the study periods will be calculated using a two-sided unpaired Student’s *t*-test [40,41]. Treatment effects will be evaluated using an ANOVA via a mixed-effects model, with fixed effects for the intervention (HIAT and WLC), period, and sequence (intervention by period interaction). In all models, random effects will include the participants nested within the sequence as a sampling cluster [42,43]. The models will be fitted using the restricted maximum likelihood estimation (RELM) to obtain the best fitting variance-covariance matrix. To explore the characteristics of the correlated longitudinal data, we will model the covariance using the first-order autoregressive [AR(1)] structure. Profile interaction plots with error bars will be generated to indicate group by time effects for the primary outcome (pain) and the primary mediators. Post hoc analysis using the Bonferroni correction will be performed. Hypothesis 1 will be supported if significant reductions in pain intensity and university absenteeism and a significant improvement in academic performance are reported following 12 weeks of HIAT compared with baseline measurements.

#### Hypothesis 2

The mediation effects of increased progesterone levels on pain will be assessed using the Sobel test [44]. A four-step model using linear regression will be tested. In Step 1, the regression coefficient between prostaglandin levels and pain (Path C) will be estimated. In Step 2, the regression coefficient between progesterone and prostaglandin levels (Path A) will be estimated. In Step 3, the regression coefficient between pain and prostaglandin levels, along with the mediator, progesterone levels (Path B), will be evaluated. In Step 4, the Sobel test will be used to estimate the standard error of Path AB using the formula B^2^SA^2^ + A^2^SB^2^, where A and B are the regression coefficients of Paths A and B, respectively, and SA and SB are the standard errors of Paths A and B, respectively [44]. The online Sobel test calculator (http://quantpsy.org/sobel/sobel.htm) will be used. Hypothesis 2 will be supported if the Sobel test indicates a significant (p < 0.05) mediation effect.

#### Hypothesis 3

The unadjusted mean costs and cost differences between HIAT and the usual care groups will be calculated for both total and disaggregated costs (intervention costs; healthcare utilisation costs, including healthcare services and medications utilised; and absenteeism costs) [37]. Seemingly unrelated regression (SUR) analyses will be used to estimate total cost differences (ΔC) and effect differences (ΔE). SUR analyses will be performed by adjusting for baseline demographics and health characteristics (such as pain intensity, menstrual cycle length, or the number of bleeding days) between groups [37,45]. The advantage of SUR is that two regression equations, one for ΔC and one for ΔE, are modelled simultaneously, allowing possible correlations between cost and outcome differences to be accounted for [37,46].

The EQ-5D responses will be converted into utility scores to estimate the gain or loss of quality-adjusted life-years (QUALYs). The incremental cost-effectiveness ratio (ICER) will be calculated using the formula ICER = ΔC/ΔE. The advantage of this approach is that our confidence intervals will not rely on the usual normality assumption. Uncertainty surrounding the ICER values and the 95% confidence intervals around cost differences will be estimated using bias-corrected and accelerated bootstrapping, with 5,000 replications [37]. The cost-effectiveness of the intervention groups will be geographically represented using the cost-effectiveness plane (CE). The threshold (λ) for cost-effectiveness, which represents the amount of money the country is willing to pay to gain one unit of effect (QALY), will be calculated.

We will determine whether the intervention is cost-effective by utilising two other thresholds to test the robustness of our conclusion: (1) GDP per capita (in terms of purchasing-power parity), which is the threshold suggested by the World Health Organisation Commission for Macroeconomics and Health for an intervention to be ‘very cost-effective’; and (2) the country-specific thresholds published by Woods et al. [47], based on extrapolating the opportunity costs of healthcare spending in the United Kingdom. The cost-effectiveness acceptability curve (CEAC) will be derived based on the λ value. Using the CEAC, the cost-effective treatment options will be predicted based on the different λ levels. A secondary analysis will be performed from a healthcare perspective by excluding absenteeism costs [37]. The robustness of the primary analysis will be tested using the following two sensitivity analyses: (1) repetition of the analysis, including completed participants alone; and (2) repetition of the analysis by excluding cost-outliers or data from participants with extremely high absenteeism costs [37]. Hypothesis 3 will be supported if HIAT yields cost (direct and indirect) savings and results in lower healthcare utility compared with WLC receiving usual care at assessment T2.

#### Qualitative data

An independent transcription service will provide verbatim transcriptions of the focus groups. Each participant will be assigned a code number for data entry. Transcripts will be analysed using the six phases of thematic analysis, following the guidelines described by Braun and Clarke [48]. The researchers will begin the analysis by reading and re-reading the transcribed data to familiarise themselves with the data (Phase 1) [48]. Relevant quotations that answer the research question will be independently marked as free quotations [48,49]. Next, the authors, PK and LL, will individually label the quotations with codes [49]. All codes will be discussed until consensus is reached (Phase 2). Subsequently, PK and LL will independently collate the codes into potential themes, gathering all data relevant to each potential theme (Phase 3) [48]. Phase 4 involves two levels. During Level 1, the authors will determine whether the themes work in relation to the coded data extracts by reading all collated extracts for each theme to ensure that the themes appear to form a coherent pattern [48]. For Level 2, the authors (PK and LL) will determine whether the themes work in relation to the entire data set; individual themes will be verified to ensure that the themes accurately reflect the meaning evident in the dataset as a whole [48]. The identified themes will then be defined and named according to their contents (Phase 5). The final set of themes will be confirmed as coherent and comprehensive by review of the team (Phase 6).

## Discussion

### Significance of the proposed study

This is the first study that proposes to use a mixed-methods model approach to evaluate the effectiveness of HIAT among women with primary dysmenorrhea by exploring the effects on academic performance and absenteeism, in addition to assessing the subjective experiences of participation in the exercise intervention and evaluating the cost-effectiveness of the intervention. Each effective intervention for primary dysmenorrhea has beneficial effects that act through unique mechanisms. Understanding the mediators of these interventions may lead to targeted treatments that distil the most critical change factors into maximally effective therapies. Understanding the mechanisms underlying aerobic exercise-induced analgesia in primary dysmenorrhea pain would help design future studies that could identify mediators of pain interventions for clinical improvements, which could themselves be the target of interventions.

### Strengths and limitations

The proposed study is the first to evaluate the effectiveness, cost-effectiveness, and physiological mechanisms underlying the beneficial effects of HIAT for primary dysmenorrhea. The other strengths of the proposed study include the robust methodology; the adequately powered design, which will be sufficient to detect statistical signficance; and the crossover design, which is less prone to confounding because each participant acts as her own control. Despite these strengths, the proposed study also has a limitation. The potential for a placebo effect is a constant concern in studies of pain [50], and exercise-based studies should be designed to account for potential placebo effects [51]; however, the proposed study design does not include a placebo control group. Placebo-controlled trials are widely regarded as the gold standards for testing the effectiveness of new treatments [52,53]. However, because exercise interventions have previously been demonstrated to have beneficial effects on primary dysmenorrhea [54], we did not consider the inclusion of a placebo control group in this study.

## Supporting information

S1 Checklist SPIRIT 2013 checklist: Recommended items to address in a clinical trial protocol and related documents*. (DOC)

S1 File Background of research research plan and methodology. (DOCX)
