# Supplementary material for: A mixed-methods study to evaluate the effectiveness and cost-effectiveness of aerobic exercise for primary dysmenorrhea: A study protocol
Source: PLoS One. 2021 Aug 16;16(8):e0256263. doi: 10.1371/journal.pone.0256263 (PMC8366973; doi:10.1371/journal.pone.0256263)
Supplement: S1 File — (DOCX) [file pone.0256263.s002.docx]

**Background of Research**

*Prevalence and impacts of dysmenorrhea****.*** Primary dysmenorrhea is a debilitating condition that affects nearly half of all menstruating women worldwide.^1^ A cross-sectional survey performed in 2013 in Hong Kong (HK) found that out of 240 18–25-year-old female university students, the prevalence of dysmenorrhea was 80%.^2^ Primary dysmenorrhea has been linked to regular educational absenteeism,^3^ and the class attendance rate among women has been reported to decrease by 29%–50% during menstruation.^4^ Recurrent absenteeism has negative impacts on young women, decreasing the total contact time for learning, which may affect the quality of the educations they receive.^3^ Studies that have examined the impacts of primary dysmenorrhea on daily life have reported that adolescent women with primary dysmenorrhea experience the reduced ability to concentrate, educational disruptions,^2^ and lower academic performance.^5^ The effects of primary dysmenorrhea-associated pain extend beyond individual women to society, resulting in regular work absenteeism.^5^

*Pathophysiology of primary dysmenorrhea.* The pathogenesis of primary dysmenorrhea has been linked to abnormally elevated prostaglandin secretion.^1, 6^ Abnormally high prostaglandin levels cause frequent and dysrhythmic uterine contractions, resulting in ischemia and hypoxia, which are considered to be major contributors to primary dysmenorrhea-associated pain.^1, 7^ Aetiological studies have reported that prostaglandin production is regulated by progesterone with prostaglandins and progesterone displaying an inverse relationship.^1, 6, 8^

*Current understanding and knowledge gaps.* Our preliminary studies^9, 10^ have identified high-intensity aerobic training (HIAT) to be effective for decreasing pain and improving daily functioning and quality of life (QoL) among women with primary dysmenorrhea. However, no study has yet evaluated the effects of exercise, specifically HIAT, on absenteeism and academic performance among university students. The physiological mechanisms that underlie the beneficial effects of aerobic exercise-induced pain relief in primary dysmenorrhea also remain unclear. *The next critical steps in our research programme include (1) the evaluation of HIAT effects on absenteeism and academic performance among university students, (2) the identification of* *the underlying mechanisms associated with aerobic exercise-induced analgesia in primary dysmenorrhea, and (3) an economic evaluation to determine the cost-effectiveness of HIAT compared with a wait-list control group receiving usual care.*

**Preliminary studies.** Initially, we conducted a narrative review^11^ to identify the potential physiological mechanisms that might underlie the beneficial effects of physiotherapy interventions for pain relief in primary dysmenorrhea. We then performed a meta-analysis^12^ to understand the effectiveness of physiotherapy interventions on pain and QoL in women with primary dysmenorrhea. In our review,^12^ we identified preliminary, but not yet definitive evidence to support the effectiveness of aerobic exercise for primary dysmenorrhea management. Therefore, we conducted a study^10^ (*n* = 10) to evaluate the feasibility, safety, and preliminary effectiveness of an aerobic exercise intervention for primary dysmenorrhea. We found that a four-week, supervised, treadmill-based, aerobic exercise intervention had large, beneficial effects on primary dysmenorrhea-associated pain quality and intensity. Subsequently, we conducted a full randomised controlled trial (RCT, *n* = 70) to evaluate the effectiveness of a seven-month aerobic exercise intervention in women with primary dysmenorrhea.^9^ In this RCT,^9^ we found that one month of supervised treadmill-based aerobic exercise, supplemented with six months of unsupervised aerobic exercise, was effective for decreasing pain intensity and improving QoL and physical function relative to usual care.

***Pilot trial***: We conducted a pilot trial to examine the effects of HIAT on plasma biomarkers that have been implicated in the pathophysiology of primary dysmenorrhea. The pilot trial findings indicated a trend towards increased progesterone levels (***d*** = 0.36) and decreased levels of prostaglandin F2 alpha (PGF2α) metabolites (***d*** = 0.35) in the HIAT group compared with those in the control group, which suggested that aerobic exercise may affect primary dysmenorrhea pain through effects on these mediators.^8, 13^

Because our research has supported the beneficial effects of HIAT, and a recent pilot study suggested a link between HIAT and progesterone and prostaglandin levels, the proposed randomised, controlled cross-over trial will test the following three hypotheses: (1) HIAT for 12 weeks will lead to significant reductions in pain intensity and absenteeism and improve academic performance compared to baseline levels; (2) pain improvements will be mediated by increased progesterone levels, resulting in decreased PGF2α levels; (3) HIAT will display superior cost-effectiveness compared with usual care (Wait-list control [WLC]) for primary dysmenorrhea treatment.

**Scientific premises for the study hypotheses**

*Hypothesis 1****.*** Previous research has shown that primary dysmenorrhea-associated pain intensity can be reduced by 30 minutes of HIAT, performed three days a week at 70%–85% of the age-adjusted maximum heart rate (MHR).^10, 14^ The RCT performed by the PI of the current proposal showed that this amount of exercise had positive and beneficial effects on pain and QoL in women with primary dysmenorrhea.^9^ Our RCT demonstrated statistically significant improvements in primary dysmenorrhea-associated pain and the mental component of QoL, and the results surpassed the threshold for clinical significance.^9^ Therefore, we hypothesise that HIAT for 12 weeks will lead to significant reductions in pain intensity and absenteeism and improve academic performance compared to baseline values.

*Hypothesis 2.* The hormones that dominate menstrual cycles are affected by exercise. Several studies have found that exercise increases progesterone levels during the luteal phase.^15-18^ High-intensity exercise has been found to induce alterations in circulating progesterone levels in both young and premenopausal women.^19, 20^ Furthermore, exercise has been associated with a reduced prevalence of primary dysmenorrhea in several studies.^14, 21-24^ Our preliminary studies^9, 10^ also found that women who participated in HIAT reported more pain relief than no-treatment controls. Because high-intensity aerobic exercise can influence both progesterone levels and analgesic effects in dysmenorrhea, we propose that improvements in pain are mediated by increased progesterone levels and the resultant decrease in PGF2α levels. Our pilot data also support this hypothesis.

*Hypothesis 3*. An economic evaluation, from the HK societal and healthcare perspective, is necessary due to cultural diversity, economic dynamics, and country-specific healthcare policies.^25, 26^ Such an evaluation will assist in decision-making regarding cost-effective treatment options for the HK population. Given the evidence supporting the beneficial effects of HIAT from our preliminary studies and the findings of our recent pilot study, which suggested a link between HIAT and progesterone levels, we hypothesise that HIAT will be superior to WLC (receiving usual care), in terms of cost-effectiveness.

***Research design and recruitment.*** A mixed-methods study, including a cross-over RCT, and semi-structured focus groups, alongside economic evaluation, will be conducted. A Cross-over design will be adopted to minimize the effect of confounders and inter-individual variability (because each participant serves as their own control)^27^ and encourage participant enrolment in the trial.^28^ A mixed-methods approach will be considered because the combination of qualitative and quantitative methods can contribute to interdisciplinary and comprehensive research evidence.^29^ This trial will include: (1) a two-step screening phase for eligibility; (2) a baseline assessment (time-point [T1]); (3) 12 weeks of supervised treadmill training for the HIAT group; (4) post-treatment outcome assessments and economic evaluations at 12 weeks (T2); (5) a washout period for four weeks, followed by the cross-over of HIAT and WLC groups; (6) 12 weeks of supervised treadmill training for the WLC group; (7) outcome assessments at 28 weeks (T3); and (8) semi-structured focus groups at 32 weeks. Potential participants will be recruited via advertisements (study flyer) distributed at the Hong Kong Polytechnic University and the Education University of Hong Kong (please see upport letter) campuses and throughout the local community and by word-of-mouth.

***Sample size:*** G-Power analysis^30^ (3.1.9.2), set for an F-test (analysis of variance (ANOVA), was used to estimate the sample size required to reliably test the study hypotheses. We will enrol 130 participants (65 per group), allowing for a 30% attrition rate (the drop-out rate in our previous RCT^9^ with a seven-month follow-up was 20%–23%). For Hypothesis 1, the estimated sample size was 86 (43 per group). The power was set to 90%, the alpha level was 0.05, the drop-out rate was 30%, and effect-size was 0.60 (from our previous RCT^9^). For Hypothesis 2, based on estimated changes in progesterone levels from our pilot trial (***d*** = 0.36),^13^ the sample size required to detect differences in progesterone with 90% power at the 5% significance level is 100 (50 per group). Assuming a 30% drop-out rate, 130 participants (65 per group) would be required.

***Participants:*** Eligibility criteria are consistent with the guidelines for the diagnosis of primary dysmenorrhea established by the Society of Obstetrics and Gynecologists of Canada.^31^ Inclusion criteria include: (1) 18–24 years old; (2) non-pregnant; (3) having regular menstrual cycles with cycle lengths between 24 and 30 days; (4) experiencing an average menstrual pain intensity equal to or greater than 5 on a 0–10 Numerical Rating Scale (NRS); and (5) scoring low (< 600 metabolic equivalent tasks [MET]/week) on the short-form of the International Physical Activity Questionnaire (IPAQ).^32^ Exclusion criteria include: (1) the use oral contraceptive pills, hormonal therapy, or intrauterine devices; (2) the use of over-the-counter analgesics during menstruation to treat dysmenorrhea-associated pain, experience no pain relief with those analgesics and (3) participation in any formal exercise programme.

***Randomisation and blinding.*** An individual who is not involved in the study will develop a randomisation schedule (computer-generated) and prepare 130 sealed, opaque envelopes for allocation concealment. The sealed opaque envelopes will contain the group name (HIAT or WLC) and personal identification number (PIN). Following the baseline assessment, participants will select an envelope containing the details of their treatment allocation. The nature of the intervention is such that neither the participants nor the therapist can be blinded to the treatment allocation. A blinded examiner (research assistant [RA]) will assess study outcomes and collect saliva for the estimation of primary and secondary mediators. The RA will be trained to perform outcome assessments and saliva collection procedures by the PI and collaborators, respectively, during a full-day training workshop. Enzyme-linked immunosorbent assays (ELISAs) will be performed by a blinded research postgraduate student (RPg) on the saliva samples, which will be labelled using the PINs by Student Assistant #2. The student assistant will enter raw data into an Excel spreadsheet for analysis. Data analysis will be conducted in a blinded manner, using coded groups (Group A and Group B). The group code will be revealed only after the analyses have been completed.

***Procedure:*** Potential participants will undergo a two-step screening process. The first step will be conducted via telephone interview, using an eligibility checklist. Potentially eligible women will then be invited to an in-person screening session on the first day of their next menstrual period. During this session, women will complete the screening questionnaire and IPAQ to determine eligibility, assess baseline physical-activity levels, and obtain written informed consent and anthropometric measurements. Baseline assessments of primary and secondary outcomes and the costs and absenteeism data associated with each individual’s previous three menstrual cycles will be performed, and saliva samples will be collected. Participants will then be randomised into HIAT or WLC groups. Participants in the HIAT group will receive supervised treadmill training for the first 12 weeks of the trial. To minimise carryover effects, a four-week washout period will be observed for all study participants before cross-over. During the washout period, the HIAT group will discontinue exercise, and both groups will continue to manage pain as usual (i.e. with analgesics). After the four-week washout period, the cross-over period will begin during Week 16, and the participants initially randomised to the WLC group will receive the HIAT regimen from Weeks 14–28. Participants initially randomized to HIAT group will be instructed not to engage in exercise during the remainder of the study period (i.e. Weeks 14-28). Participants will be permitted to use analgesics for menstrual pain as needed throughout the study and will be instructed to record the dose and number of pills consumed in an electronic diary. As a diagnosis of primary dysmenorrhea can be confirmed by collecting detailed history;^33, 34^ a pelvic examination/ ultrasound will not be conducted.

***Saliva collection*.** Saliva will be collected as described in a previous study.^35^ All participants will receive salivary kits and specimen bag with participant details [name, PIN and group allocation]) and written instructions for saliva collection procedures. The RA will provide training to all participants for the collection of citric-acid stimulated saliva (approximately 5 mL). In our recent pilot trial, mediators levels were evaluated in plasma; however, in the current proposed study, these mediators will be measured in saliva. Some participants from our recent pilot trial, in which we collected plasma for the estimation of mediator levels,^13^ reported a preference for non-invasive diagnostic tests over invasive tests (blood collection) and expressed difficulty reporting to PolyU for blood collection when their menstrual periods started on weekends. Additionally, the circulating levels of PGF_2α_ in peripheral plasma are commonly very low because PGF_2α_ is inactivated during its initial entry through the lungs.^36^ Studies have reported that salivary measures provide a reliable and non-invasive method for assessing progesterone^37^ and prostaglandins.^35^ The home collection of saliva is convenient and cost-effective and can be done easily and safely without assistance.^38^ Therefore, for the current proposed study, mediators concentrations will be measured in the saliva, instead of blood plasma. Participants will be instructed to collect saliva immediately after the start of menstruation and two hours following the start of menstruation, to store samples in a freezer.^35^ The RA will transport the samples to the lab for further processing.

***Intervention.*** The exercise intervention will last for 24 weeks (12 weeks for the HIAT group and 12 weeks for the WLC group) at PolyU. Supervised treadmill (Zebris FDM-T) training at PolyU will begin on the first day of the second menstrual period (baseline assessments will be completed during the first menstrual period). Exercise sessions at PolyU will be instructed and supervised by a registered physiotherapist (RPg student). Using this supervisory method, the participant-to-research person ratio will be 1:1. The rationale for providing supervised sessions is to increase adherence to study interventions because, in our previous RCT^9^, adherence was higher for supervised sessions than for unsupervised home exercise sessions. MHR will be calculated using the conventional age-predicted formula (220 – age). To achieve the target HR, the speed of the treadmill will be gradually increased, based on the participants’ HR, which will be displayed on the treadmill, and the rating of perceived exertion (RPE). Because the current proposed study does not involve graded, incremental exercise, graded-exercise testing (such as VO_2max_ evaluation) will not be performed.

*HIAT.* Women will perform treadmill-based aerobic exercise for three days a week, at 70%–85% of MHR for 30 minutes and perceived exertion of 14–16, based on the Borg RPE scale. This range^39^ is considered to represent HIAT. Aerobic training will be preceded by warm-up exercises for 10 minutes and followed by cool-down exercises for 10 minutes, at a perceived exertion of 11.0 (Borg RPE). These exercise parameters are based on our previous studies^9,10^ and the American College of Sports Medicine guidelines.^40^

*WLC*. Women in the WLC group will be instructed to continue with their usual activities and manage their pain as normal (i.e., with analgesics).

**Outcome measures: Primary outcomes:** *Pain intensity.* Average pain intensity during the last 24 hours during menstruation will be assessed using the 0–10 NRS,^41^ with 0 representing ‘No pain’ and 10 representing ‘Worst imaginable pain’.

*Absenteeism from university*: Access to a prospective electronic diary will be provided for each participant to record university absenteeism. Electronic diaries are reported as highly acceptable and feasible to use.^44^ Significantly increased compliance and accuracy have been reported with e-diary recording compared with traditional paper diaries.^44^

*Academic performance*: Academic performance will be measured using the self-reported 20-item academic performance questionnaire, which was developed to measure the impacts of menstrual symptoms on academic performance.^45^

**Primary mediators:** *Salivary progesterone and PGF2α levels*. Salivary measures provide a reliable and non-invasive method for assessing unbound steroid hormones in humans.^37^ Salivary progesterone levels have been significantly correlated with plasma levels during the menstrual cycle and reflect free serum progesterone levels.^37^ Previous studies have identified elevated levels of PGF2α in saliva during menstruation^35^ and reported that saliva could be used as an important source of biomarkers, such as PGF2α.^42^

**Secondary outcomes:** *Concentration:* The 0–10 visual analogue scale will be used to measure the impact of dysmenorrhea on concentration,^43^ with 0 representing ‘No difficulty concentrating’ and 10 representing ‘Maximum difficulty concentrating’.

*Dysmenorrhea daily diary (DysDD)*: The 10-item, disease-specific DysDD (electronic version) measures the severity of menstrual bleeding, the use of analgesic for pelvic pain, and the impacts of menstrual pain on work/education, physical and social activities, and sleep.^46^ The DysDD has been reported to be an adequately reliable and valid tool for assessing dysmenorrhea.^47^ To minimise burden, participants will be asked to complete the DysDD only on days when their pain rating is non-zero or when rescue analgesics are taken.^46^

*Economic evaluation*. We will analyse the results of HIAT versus WLC over a 12-week, within-trial period (i.e., at T2).^28^ The cost-effectiveness for the entire 28-week time horizon will also be explored through T3 data observed in the HIAT arm and extrapolation of WLC data.^28^ The primary analysis will be conducted from a societal perspective (short-term direct costs, intervention costs, costs unrelated to the intervention [healthcare services and medication costs] and indirect costs, such as absence from work and impact on productivity due to primary dysmenorrhea).^48^ The secondary analysis will be conducted from a healthcare perspective (direct costs, including intervention costs and costs of other care, *excluding* absenteeism costs).^48^ Cost and absenteeism data that are collected at baseline (i.e., previous three menstrual cycles) will be used as predictors of future costs in the analysis. The EQ-5D-3L will be used to determine the QoL for the HIAT and WLC groups. The cost per participant will be calculated by multiplying unit costs identified from the HK government standardised national price list and resource quantities.^28^

An electronic diary (<https://forms.gle/gvoLHAoDqySQFr4GA>) will be used to record cost expenditures and work absenteeism. All participants will be provided with access to the cost diary, post-randomisation. Participants with limited access to the internet will be provided with printed hard copies of the cost and absenteeism diary, along with stamped and addressed return envelopes. Participants will be instructed to complete the diary during the week of menstruation each month. The research team members will be able to access the electronic diary to obtain a summary of the completed items for all study participants. The student assistant will contact participants by telephone for incomplete entries, to acquire additional details, or for failure to complete the diary.

**Qualitative study (semi-structured focus groups):** The qualitative study will provide insight into the participants’ experiences during and attitudes towards the study intervention. During focus group discussions, attempts will be made to identify the subjective experiences with participation in HIAT and participants’ perceived improvements in menstrual pain, university attendance, and academic performance. For this component of the research, a computer-generated randomisation schedule will be generated and utilised to identify 70 participants (from the 130 participants included in the RCT), to participate in the focus groups. Ethics approval and written informed consent will be obtained before enrolment. A total of seven focus groups will be conducted at 32 weeks post-randomisation. Each group will consist of a random sample of 8-10 participants and will last between 30 and 45 minutes. Focus groups will be conducted in private rooms at PolyU. If the coronavirus disease 2019 pandemic continues, face-to-face seminars will be replaced with virtual meetings (Zoom) to allow appropriate social distancing. Two researchers, a moderator (PI) and a co-moderator (RPg student), will conduct the focus groups, and a third research team member will take notes. The interviews will be recorded using a digital voice recorder, in addition to taking notes. Semi-structured discussion guides will be used.

***Treatment fidelity and missed sessions.*** To optimise adherence to exercise sessions at PolyU, a student assistant will contact any participant who has missed an exercise session and attempt to re-schedule that session. Following the baseline assessment, all participants will be provided with information regarding how to access and complete the electronic diaries.

***Retention plan.*** To maximise retention we: (1) will obtain the contact information of participants and any friends and relatives who may know their whereabouts, and obtaining permission to follow-up the listed contact persons; (2) include appointments for data collection in the study schedule; (3) schedule supervised exercise at convenient times; and (4) offer a remuneration of HK$ 200 to cover the costs incurred during study participation.

**Statistical tests and anticipated results**

*Missing data and data preparation*. We will implement strategies, such as the digital diary, follow-up phone-call reminders, and providing routine encouragement to continue exercise, to minimise missing data. Missing values will be replaced with group means. All statistical analyses will be performed on an intention-to-treat basis, using SPSS (version 24).

***Hypothesis 1.*** The two phases that each participant completes during the course of a cross-over trial are typically referred to as the two study periods.^49^ First, a preliminary test will be carried to rule out carryover effects and to test our study for validity.^50^ This will be performed by estimating the sum of the values measured during the two treatment periods for each participant and compared across the groups by using an independent samples *t*-test.^49^ Second, the within-participant differences in outcome variables between the study periods will be calculated using a two-sided Student’s unpaired *t*-test.^49, 50^ Treatment effects will be evaluated using an ANOVA with a mixed-effects model considering the longitudinal repeated measures, including the effects of time (T1, T2, and T3) within each study group and the interaction between time and intervention. Post hoc analysis using the Bonferroni correction will be performed. Hypothesis 1 will be supported if significant reductions in pain intensity and university absenteeism and a significant improvement in academic performance are reported following 12 weeks of HIAT compared with baseline measurements.

***Hypothesis 2.*** The mediation effects of increased progesterone levels on pain will be assessed using the Sobel test.^51^ A four-step model using linear regression will be tested. In Step 1, the regression coefficient between prostaglandin levels and pain (Path C) will be estimated. In Step 2, the regression coefficient between progesterone and prostaglandin levels (Path A) will be estimated. In Step 3, the regression coefficient between pain and prostaglandin levels, along with the mediator, progesterone levels (Path B), will be evaluated. Step 4: the Sobel test will be used to estimate the standard error of Path AB using the formula B^2^SA^2^ + A^2^SB^2^, where A and B are the regression coefficients of Paths A and B, respectively, and SA and SB are the standard errors of Paths A and B, respectively.^51^ The online Sobel test calculator (<http://quantpsy.org/sobel/sobel.htm>) will be used. Hypothesis 2 will be supported if the Sobel test indicates a significant (p < 0.05) mediation effect.

***Hypothesis 3***. The unadjusted mean costs and cost differences between HIAT and the usual care groups for total and disaggregated costs (intervention costs, healthcare utilisation costs, including healthcare services and medications utilised, and absenteeism costs) will be calculated.^48^ The seemingly unrelated regression (SUR) analyses will be used to estimate the total cost differences (ΔC) and effect differences (ΔE). The SUR analyses will be performed by adjusting for baseline demographic and health characteristics (such as pain intensity and menstrual-cycle length or the number of bleeding days) between groups.^48, 52^ The advantage of SUR is that two regression equations, one for ΔC and one for ΔE, are modelled simultaneously allowing possible correlations between cost and outcome differences to be accounted for.^48, 53^

The EQ-5D responses will be converted into utility scores to estimate the gain or loss of quality-adjusted life-years (QUALYs). The incremental cost-effectiveness ratio (ICER) will be calculated using the formula ICER = ΔC / ΔE. The advantage of this approach is that our confidence intervals do not rely on the usual normality assumption. Uncertainty surrounding the ICERs and the 95% confidence intervals around cost differences will be estimated using bias-corrected and accelerated bootstrapping with 5,000 replications.^48^ The cost-effectiveness of the intervention groups will be geographically represented using the cost-effectiveness plane (CE). The threshold (λ) for cost-effectiveness or the amount of money the country is willing to pay to gain one unit of effect (QALY) will be calculated.

Further, we will determine whether the intervention is cost-effective by utilising two other thresholds to test the robustness of our conclusion: (1) GDP per capita (in terms of purchasing-power parity), which is the threshold suggested by the World Health Organisation Commission for Macroeconomics and Health for an intervention to be ‘very cost-effective’;^54^ and (2) the country-specific thresholds published by Woods et al.,^55^ based on extrapolating the opportunity costs of healthcare spending in the United Kingdom. The cost-effectiveness acceptability curve (CEAC) will be derived based on the λ value. Using the CEAC, the cost-effective treatment options will be predicted based on the different λ levels. A secondary analysis will be performed from a healthcare perspective by excluding absenteeism costs.^48^ The robustness of the primary analysis will be tested using the following two sensitivity analyses: (1) repetition of the analysis including completed participants alone; and (2) excluding cost-outliers or data from participants with extremely high absenteeism costs.^48^ Hypothesis 3 will be supported if HIAT yields cost (direct and indirect) savings and results in lower healthcare utility compared with WLC receiving usual care at assessment T2.

***Qualitative data***: An independent transcription service will provide verbatim transcriptions of the focus groups. Participants’ anonymity will be maintained by replacing names with PINs. Transcripts will be compared with audio recordings to ensure data accuracy. Transcripts will be analysed using the process of thematic analysis, as described by Braun and Clarke.^56^ The PI will initially analyse the transcripts from each focus group individually and then compare emergent themes across all focus groups. The final set of themes will be confirmed as coherent and comprehensive by the collaborators.

1. Dawood MY. Primary dysmenorrhea: advances in pathogenesis and management. Obstetrics & Gynecology. 2006;108(2):428-41.

2. Chia C, Lai J, Cheung P, Kwong L, Lau F, Leung K, et al. Dysmenorrhoea among Hong Kong university students: prevalence, impact, and management. Hong Kong Med J. 2013;19(3):222-8.

3. Femi-Agboola DM, Sekoni OO, Goodman OO. Dysmenorrhea and its effects on school absenteeism and school activities among adolescents in selected secondary schools in Ibadan, Nigeria. Nigerian medical journal: journal of the Nigeria Medical Association. 2017;58(4):143.

4. Sundell G, Milsom I, ANDERSCH B. Factors influencing the prevalence and severity of dysmenorrhoea in young women. BJOG: An International Journal of Obstetrics & Gynaecology. 1990;97(7):588-94.

5. Bernardi M, Lazzeri L, Perelli F, Reis FM, Petraglia F. Dysmenorrhea and related disorders. F1000Research. 2017;6.

6. Dawood MY. Dysmenorrhea and prostaglandins. Gynecologic endocrinology: Springer; 1987. p. 405-21.

7. Proctor M, Farquhar C. Diagnosis and management of dysmenorrhoea. BMJ. 2006;332(7550):1134-8.

8. Kannan P, Cheung K-K, Lau BW-M. Does aerobic exercise induced-analgesia occur through hormone and inflammatory cytokine-mediated mechanisms in primary dysmenorrhea? Medical hypotheses. 2019;123:50-4.

9. Kannan P, Chapple CM, Miller D, Claydon-Mueller L, Baxter GD. Effectiveness of a treadmill-based aerobic exercise intervention on pain, daily functioning, and quality of life in women with primary dysmenorrhea: A randomized controlled trial. Contemporary clinical trials. 2019;81:80-6.

10. Kannan P, Claydon LS, Miller D, Chapple CM. Vigorous exercises in the management of primary dysmenorrhea: a feasibility study. Disability & Rehabilitation. 2014(0):1-6.

11. Kannan P, Claydon LS. Physiological rationales of physical therapy interventions in the management of primary dysmenorrhea: a critical review. Physical Therapy Reviews. 2015;20(2):98-109.

12. Kannan P, Claydon LS. Some physiotherapy treatments may relieve menstrual pain in women with primary dysmenorrhea: a systematic review. Journal of physiotherapy. 2014;60(1):13-21.

13. Kannan P, Cheung K-K, Chi CS, Yan LK, Xi LY, Ki NS, et al. Effect of Aerobic Exercise on Plasma Biomarkers of Pain in Women with Primary Dysmenorrhea: A Controlled Non-Randomized Pilot Trial. Biomarkers. 2020;6(2).

14. Israel RG, Sutton M, O'Brien KF. Effects of aerobic training on primary dysmenorrhea symptomatology in college females. Journal of American College Health. 1985;33(6):241-4.

15. Bonen A, Haynes F, Watson-Wright W, Sopper M, Pierce G, Low M, et al. Effects of menstrual cycle on metabolic responses to exercise. Journal of Applied Physiology. 1983;55(5):1506-13.

16. Kraemer RR, Heleniak RJ, Tryniecki JL, Kraemer GR, Okazaki NJ, Castracane VD. Follicular and luteal phase hormonal responses to low-volume resistive exercise. Medicine and science in sports and exercise. 1995;27(6):809-17.

17. Otağ A, Turaçlar TU, Otağ İ. Evaluation of body composition and basal metabolic rate after acute exercise in menstrual phases in sportswomen. Cumhuriyet Medical Journal. 2011;33(1):53-60.

18. Williams NI, Bullen BA, McARTHUR JW, Skrinar GS, Turnbull BA. Effects of short-term strenuous endurance exercise upon corpus luteum function. Medicine and science in sports and exercise. 1999;31(7):949-58.

19. Bonen A, Ling W, MacIntyre K, Neil R, McGrail J, Belcastro A. Effects of exercise on the serum concentrations of FSH, LH, progesterone, and estradiol. European journal of applied physiology and occupational physiology. 1979;42(1):15-23.

20. Atuegbu CM, Meludu SC, Dioka CE, Onyenekwe CC, Onuegbu JA, Onah CE, et al. Effect of moderate-vigorous intensity physical exercise on female sex hormones in premenopausal university students in Nnewi, Nigeria. International Journal of Research in Medical Sciences. 2017;2(4):1516-20.

21. Aganoff JA, Boyle GJ. Aerobic exercise, mood states and menstrual cycle symptoms. Journal of psychosomatic research. 1994;38(3):183-92.

22. Dehghanzadeh N, Khoshnam E, Nikseresht A. The effect of 8 weeks of aerobic training on primary dysmenorrhea. European Journal of Experimental Biology. 2014;4(1):380-2.

23. Lee I-M. Dose-response relation between physical activity and fitness: even a little is good; more is better. Jama. 2007;297(19):2137-9.

24. Mahvash N, Eidy A, Mehdi K, Zahra MT, Mani M, Shahla H. The effect of physical activity on primary dysmenorrhea of female university students. World Applied Sciences Journal. 2012;17(10):1246-52.

25. Group TE. EuroQol-a new facility for the measurement of health-related quality of life. Health policy. 1990;16(3):199-208.

26. Winser S, Lee SH, Law HS, Leung HY, Bello UM, Kannan P. Economic evaluations of physiotherapy interventions for neurological disorders: a systematic review. Disability and rehabilitation. 2020;42(7):892-901.

27. Mills EJ, Chan A-W, Wu P, Vail A, Guyatt GH, Altman DG. Design, analysis, and presentation of crossover trials. Trials. 2009;10(1):27.

28. Doble B, Langdon PE, Shepstone L, Murphy GH, Fowler D, Heavens D, et al. Economic Evaluation Alongside a Randomized Controlled Crossover Trial of Modified Group Cognitive–Behavioral Therapy for Anxiety Compared to Treatment-as-Usual in Adults With Asperger Syndrome. MDM policy & practice. 2017;2(2):2381468317729353.

29. Rauscher L, Greenfield BH. Advancements in contemporary physical therapy research: use of mixed methods designs. Physical Therapy. 2009;89(1):91-100.

30. Erdfelder E, Faul F, Buchner A. GPOWER: A general power analysis program. Behavior research methods, instruments, & computers. 1996;28(1):1-11.

31. Lefebvre G, Pinsonneault O, Antao V, Black A, Burnett M, Feldman K, et al. Primary dysmenorrhea consensus guideline. J Obstet Gynaecol Can. 2005;27(12):1117-46.

32. Committee IR. Guidelines for data processing and analysis of the International Physical Activity Questionnaire (IPAQ)–short and long forms. Retrieved September. 2005;17:2008.

33. Burnett MA, Antao V, Black A, Feldman K, Grenville A, Lea R, et al. Prevalence of primary dysmenorrhea in Canada. Journal of obstetrics and gynaecology Canada: JOGC= Journal d'obstetrique et gynecologie du Canada: JOGC. 2005;27(8):765-70.

34. Durain D. Primary dysmenorrhea: assessment and management update. Journal of Midwifery & Women’s Health. 2004;49(6):520-8.

35. Durham PL, Vause CV, Derosier F, McDonald S, Cady R, Martin V. Changes in salivary prostaglandin levels during menstrual migraine with associated dysmenorrhea. Headache: The Journal of Head and Face Pain. 2010;50(5):844-51.

36. Piper PJ, Vane J, Wyllie J. Inactivation of prostaglandins by the lungs. Nature. 1970;225(5233):600.

37. Kaufman E, Lamster IB. The diagnostic applications of saliva—a review. Critical Reviews in oral biology & medicine. 2002;13(2):197-212.

38. Liu J, Duan Y. Saliva: a potential media for disease diagnostics and monitoring. Oral oncology. 2012;48(7):569-77.

39. Norton K, Norton L, Sadgrove D. Position statement on physical activity and exercise intensity terminology. Journal of Science and Medicine in Sport. 2010;13(5):496-502.

40. Pescatello LS, Riebe D, Thompson PD. ACSM's guidelines for exercise testing and prescription: Lippincott Williams & Wilkins; 2014.

41. Hawker GA, Mian S, Kendzerska T, French M. Measures of adult pain: Visual analog scale for pain (vas pain), numeric rating scale for pain (nrs pain), mcgill pain questionnaire (mpq), short‐form mcgill pain questionnaire (sf‐mpq), chronic pain grade scale (cpgs), short form‐36 bodily pain scale (sf‐36 bps), and measure of intermittent and constant osteoarthritis pain (icoap). Arthritis care & research. 2011;63(S11):S240-S52.

42. Prasad S, Tyagi AK, Aggarwal BB. Detection of inflammatory biomarkers in saliva and urine: potential in diagnosis, prevention, and treatment for chronic diseases. Experimental Biology and Medicine. 2016;241(8):783-99.

43. Orhan C, Çelenay ŞT, Demirtürk F, Özgül S, Üzelpasacı E, Akbayrak T. Effects of menstrual pain on the academic performance and participation in sports and social activities in Turkish university students with primary dysmenorrhea: A case control study. Journal of Obstetrics and Gynaecology Research. 2018;44(11):2101-9.

44. Palermo TM, Valenzuela D, Stork PP. A randomized trial of electronic versus paper pain diaries in children: impact on compliance, accuracy, and acceptability. Pain. 2004;107(3):213-9.

45. Raju J, Suguna M. A study to assess the effect of menstrual symptoms on academic performance among nursing students at selected colleges in Tamil Nadu, India. IJAR. 2017;3(3):78-80.

46. Nguyen AM, Humphrey L, Kitchen H, Rehman T, Norquist JM. A qualitative study to develop a patient-reported outcome for dysmenorrhea. Quality of Life Research. 2015;24(1):181-91.

47. Nguyen AM, Arbuckle R, Korver T, Chen F, Taylor B, Turnbull A, et al. Psychometric validation of the dysmenorrhea daily diary (DysDD): a patient-reported outcome for dysmenorrhea. Quality of Life Research. 2017;26(8):2041-55.

48. Williams A, van Dongen JM, Kamper SJ, O'Brien KM, Wolfenden L, Yoong SL, et al. Economic evaluation of a healthy lifestyle intervention for chronic low back pain: A randomized controlled trial. European Journal of Pain. 2019;23(3):621-34.

49. Wellek S, Blettner M. On the proper use of the crossover design in clinical trials: part 18 of a series on evaluation of scientific publications. Deutsches Ärzteblatt International. 2012;109(15):276.

50. Sczesny-Kaiser M, Trost R, Aach M, Schildhauer TA, Schwenkreis P, Tegenthoff M. A randomized and controlled crossover study investigating the improvement of walking and posture functions in chronic stroke patients using HAL exoskeleton–The HALESTRO study (HAL-Exoskeleton STROke Study). Frontiers in neuroscience. 2019;13:259.

51. Preacher KJ, Leonardelli GJ. Calculation for the Sobel test. Retrieved January. 2001;20:2009.

52. Srivastava VK, Giles DE. Seemingly unrelated regression equations models: Estimation and inference: CRC press; 2020.

53. Willan AR, Briggs AH, Hoch JS. Regression methods for covariate adjustment and subgroup analysis for non‐censored cost‐effectiveness data. Health economics. 2004;13(5):461-75.

54. Feachem RG. Commission on macroeconomics and health. SciELO Public Health; 2002.

55. Woods B, Revill P, Sculpher M, Claxton K. Country-level cost-effectiveness thresholds: initial estimates and the need for further research. Value in Health. 2016;19(8):929-35.

56. Braun V, Clarke V. Using thematic analysis in psychology. Qualitative research in psychology. 2006;3(2):77-101.
